# Risk-adapted intervals for colorectal cancer screening using fecal immunochemical tests: a modelling study based on the BLITZ cohort

**DOI:** 10.1186/s12916-026-05138-7

**Published:** 2026-08-18

**Authors:** Thomas Heisser, Teresa Seum, Michael Hoffmeister, Hermann Brenner

**Affiliations:** 1 Cancer Prevention Graduate School, German Cancer Research Center (DKFZ), Heidelberg, Germany; 2 Division of Clinical Epidemiology and Aging Research, German Cancer Research Center (DKFZ), Heidelberg, Germany; 3 Heidelberg Medical Faculty, Heidelberg University, Heidelberg, Germany

**Keywords:** Colorectal cancer, Fecal immunochemical testing, Screening, Modelling

## Abstract

**Background:**

Established screening programs for colorectal cancer (CRC) based on fecal immunochemical tests (FITs) employ fixed screening intervals of either one year or two years for all participants. We aimed to assess the potential design of risk-adapted FIT screening intervals based on quantitative values of the fecal hemoglobin concentration among FIT negative participants.

**Methods:**

Using COSIMO, a previously validated simulation tool, we compared CRC risks in subgroups with initially negative FIT. Subgroups were stratified by stool hemoglobin levels at screening (< 8 (low), 8-<10 (medium), 10-<17 µg (high) Hb/g stool), a routinely available measure in FIT-based screening programs. Colorectal neoplasia prevalences at model start were informed by 6,661 screening colonoscopy participants in Germany at average CRC risk with negative FIT and available colonoscopy results (BLITZ study). Simulations were run for hypothetical cohorts of 100,000 individuals.

**Results:**

Of 6,661 FIT-negative participants, 6,016 (90%), 273 (4%), and 372 (6%) were in the low, medium, and high-negative subgroups, respectively. In those low-negative, expected cancer detection rates were 0.17%, 0.30%, 0.42%, 0.53%, and 0.62%, after screening intervals of 1, 2, 3, 4, and 5 years, respectively. Cancer detection rates among the small groups of medium- and high-negative patients were up to six times higher and exceeded the expected 2-year detection rates of the low-negative group already after 1 year of follow-up.

**Conclusions:**

Fecal hemoglobin concentrations among FIT negative participants may be a readily available, highly informative tool for defining risk-adapted annual or biennial FIT screening intervals.

**Supplementary Information:**

The online version contains supplementary material available at 10.1186/s12916-026-05138-7.

## Background

Colorectal cancer (CRC) is one of few cancers for which effective screening options are established [1]. A broadly recommended and increasingly popular option is screening by fecal immunochemical tests (FITs), a modern stool test [2, 3]. FITs provide a safe, non-invasive, easy-to-use option at modest costs [4]. However, the intervals after which a negative FIT should be repeated is subject to discussion. Whereas annual FIT screening is recommended in the United States (US) [5], biennial FIT screening is recommended and offered across many European countries [6]. In reality, both intervals will likely be adequate, but for different groups of participants. However, it remains unclear how to select patients who will benefit most from annual or biennial intervals.

Such risk stratification could possibly exploit the quantitative nature of FITs. FITs are currently used in a qualitative, binary fashion, largely ignoring their potential to exactly quantify fecal hemoglobin (hb) concentrations. Subjects with hemoglobin levels above a certain cutoff (i.e., a ‘positive’ test), are recommended for diagnostic work-up, while those with levels below cutoff (i.e., a ‘negative’ test) are reinvited for testing at uniform intervals. However, the quantitative measure of hemoglobin below the pre-defined positivity cutoff might be a suitable candidate for more refined risk stratification among FIT negative screenees. Recent evidence showed strong variation of advanced neoplasia prevalence according to the quantitative FIT result among those with a (qualitatively) ‘negative’ FIT outcome. Specifically, in individuals with a negative result of FOB Gold, a commercial FIT with a recommended positivity threshold at 17 µg Hb/g stool, prevalence of advanced neoplasia ranged from ≤ 6% among approximately 90% of FIT negative participants with a hemoglobin concentration < 8 µg/g to 10–22% among a minority of approximately 10% of FIT negative participants with a FIT value in the range of 8–17 µg/g [7].

Such strong variation likely implies that those in the upper range of negative FIT values are at higher risk of developing CRC than those in the lower range. Consequently, risk-adapted FIT screening intervals, with annual intervals for those in the upper range and biennial intervals for those in the lower range, might be a superior strategy to uniform screening intervals. Specifically, a more differentiated, risk-stratified approach might help to detect a higher share of advanced neoplasms in the high-risk groups, contributing to reducing CRC incidence and mortality, while avoiding premature repeated (over-)screening in those at low risk. In this modelling study, we assessed the potential design of such risk-adapted FIT screening intervals.

## Methods

### Multistate markov model

To derive and explore potential risk-adapted FIT screening intervals, we used the *Co*lorectal Cancer Multistate *Si*mulation *Mo*del (COSIMO), a validated, Markov-based simulation tool [8], to simulate a hypothetical German cohort of screening participants with negative FIT result at baseline. Briefly, COSIMO simulates the natural history of CRC based on the process of precursor lesions developing into preclinical and then clinical cancer for a predefined number of years. Screening can interfere with the natural history of CRC (Additional File 1: Supplementary Fig. 1).

The model’s natural history assumptions were derived step-by-step in several previous analyses using data from the German screening colonoscopy registry, the world’s largest registry of its kind [9–11]. Death rates from CRC were estimated using data from a large population-based case-control study with long-term follow-up of CRC cases and registry data from Germany [12, 13]. General mortality rates and average life expectancy were extracted from German population life Table [14].

A comprehensive documentation of the model’s structure and data sources used for its development is given in Additional File 1: Supplementary Methods [9–27]. Overviews of key model parameters are provided in Additional File 1: Supplementary Tables 1–3. The model source code, developed in the statistical software R, is available for download from our website [24]. R version 4.5.2 was used for all analyses.

### Sex- and age-specific baseline prevalences

To inform adenoma and cancer prevalences of screening participants with negative FIT results at model start (i.e. baseline), we used data of the BLITZ (Begleitende Evaluierung innovativer Testverfahren zur Darmkrebsfrüherkennung) study. Briefly, BLITZ is an ongoing study in screening colonoscopy participants recruited through 20 gastroenterology practices in Southern Germany with the primary aim to evaluate novel stool and blood tests for CRC screening. More details on the BLITZ study design have previously been reported elsewhere [28–30]. For this study, in line with a previous analysis [7], we derived adenoma and cancer prevalences among participants of the German screening colonoscopy program recruited in the BLITZ study who conducted a quantitative FIT (FOB Gold, Sentinel Diagnostics, Milan, Italy) prior to colonoscopy and who had a FIT result below 17 µg Hb/g stool, i.e., the cutoff recommended by the manufacturer. For this analysis, colonoscopy results were categorized according to the most advanced finding, and the reported prevalence is the percentage of individuals with the corresponding most advanced finding at colonoscopy. Prevalences were stratified by FIT negative subgroup (< 8, 8-<10, 10-<17 µg Hb/g stool) as identified by a previous analysis [7]. While in the previous analysis, in total six strata were reported < 1.7, 1.7-<8, 8-<9, 9-<10, 10-<12, 12-<17), we limited the current analyses to only three strata to allow for a pragmatic risk-adapted approach viable for implementation in real-life screening practice.

Participants were selected from 10,061 participants undergoing colonoscopy for primary screening recruited between November 2008 and December 2020. After applying pre-specified exclusion criteria as described in previous reports [28–30] and Additional File 1: Supplementary Methods, 6,661 participants were included in the analysis (Additional File 1: Supplementary Fig. 2). Baseline characteristics of the cohort are provided in Additional File 1: Supplementary Table 4**.** An overview of sex-specific baseline prevalences (proportions and 95% confidence intervals) by FIT negative subgroups is given in Table 1. All participants provided written informed consent. The BLITZ study was approved by the ethics committees of Heidelberg University and the state medical chambers of Baden-Württemberg, Saarland, Rhineland Palatinate, and Hesse.

### Simulations

To derive potential risk-adapted FIT screening intervals by FIT negative group, we simulated natural history models (i.e., assuming no further screening intervention) for five years of follow-up in sex-specific subgroups for cohorts of 100,000 subjects for each FIT negative stratum. The model was run separately for each stratum using the sex- and age-specific baseline prevalences at model start as derived from the BLITZ data as described above. Transition rates between states were assumed consistent across FIT negative strata. As currently uniform screening intervals are recommended for both sexes, sex-specific analyses were combined to reflect the overall screen-eligible population. The model starting age was set to 60 years, in line with median age of the included BLITZ cohort [7].

### Outcomes

We assessed the expected prevalence of colonoscopy-detectable preclinical cancers and any advanced neoplasms (preclinical cancers and advanced adenomas) after 1, 2-, 3-, 4-, or 5-years screening intervals by calculating the expected detection rate of a hypothetical colonoscopy at these timepoints. Almost perfect detectability by colonoscopy of 95% of advanced lesions was assumed, in line with estimates reported in the literature [26] and as used in previous simulations [8].

Note that the detection rates of preclinical cancers and any advanced neoplasms derived in this way reflect a snapshot of the respective prevalences at a given timepoint (e.g., 1–5 years after first screening). However, precursor lesions may progress to manifest cancers at a certain probability within longer screening intervals. As secondary outcome, we therefore calculated the cumulative interval cancer incidence (as opposed to cancers detected by screening) over time if screening intervals were extended beyond 2 years in the subgroup of participants with low-negative FIT results. As compared to the expected prevalences at screening, the cumulative interval cancer incidence assesses the proportion of individuals with cancers that have become manifest before screening took place.

### Patient and public involvement

Patients and the public were neither involved in the design and conduct of this study, nor in writing or editing of this document. Research at the German Cancer Research Center (DKFZ) is generally informed by a Patient Advisory Committee.

## Results

### BLITZ analysis outcomes

Observed proportions of no neoplasm and observed prevalences of non-advanced adenoma, any advanced neoplasm and cancers by FIT-negative subgroup (i.e., < 8, 8–10, and 10-<17 µg/g) as detected in 6,661 participants of the BLITZ study and used to inform the beginning of simulations are shown in Table 1. Prevalences of any advanced neoplasms increased from 5.6% among those with hemoglobin concentrations < 8 µg/g to 18.5% in the highest group of participants with FIT-negative results (10-<17 µg/g). Prevalences were highest (38.9%) in those with positive FIT (included for reference). No cancers were detected among individuals with low FIT values. Cancer prevalence was low among medium- and high-negative participants (0.4 and 0.8%, respectively), and high (6.6%) among those tested positive.

**Table 1:** Proportions of participants with no neoplasm, non-advanced adenoma and any advanced neoplasm at the beginning of simulation

Test outcome (binary)	FIT group (µg Hb/g )	Number of participants (*n*, % of all, % of all negative)	Most advanced finding % (95% confidence interval)			
			no neoplasm	non-advanced adenoma	any advanced neoplasm	cancer
		all				
negative	< 8	6016 (81%) (90%)	76.1 (75.1–77.2)	18.2 (17.3–19.2)	5.6 (5.1–6.2)	0.0 (0.0–0.0)
	8-<10	273 (4%) (4%)	67.0 (61.5–72.6)	20.9 (16.1–25.7)	12.1 (8.2–16.0)	0.4 (0.0–1.1)
	10-<17	372 (5%) (6%)	53.2 (48.2–58.3)	28.2 (23.7–32.8)	18.5 (14.6–22.5)	0.8 (0.0–1.7)
positive ^1^	≥ 17	737 (10%) (-)	43.7 (40.1–47.3)	17.4 (14.6–20.1)	38.9 (35.4–42.5)	6.6 (4.8–8.4)
		men				
negative	< 8	2766 (77%) (88%)	69.6 (67.9–71.3)	23.5 (21.9–25.0)	6.9 (6.0–7.9)	0.0 (0.0–0.0)
	8-<10	152 (4%) (5%)	58.6 (50.7–66.4)	27.6 (20.5–34.7)	13.8 (8.3–19.3)	0.7 (0.0–1.9)
	10-<17	237 (7%) (8%)	48.1 (41.7–54.5)	30.8 (24.9–36.7)	21.1 (15.9–26.3)	0.8 (0.0–2.0)
positive ^1^	≥ 17	428 (12%) (-)	36.7 (32.1–41.2)	20.3 (16.5–24.1)	43.0 (38.3–47.7)	7.0 (4.6–9.4)
		women				
negative	< 8	3250 (85%) (93%)	81.7 (80.3–83.0)	13.8 (12.6–15.0)	4.6 (3.8–5.3)	0.0 (0.0–0.1)
	8-<10	121 (3%) (3%)	77.7 (70.3–85.1)	12.4 (6.5–18.3)	9.9 (4.6–15.2)	0.0 (0.0–0.0)
	10-<17	135 (4%) (4%)	62.2 (54.0–70.4)	23.7 (16.5–30.9)	14.1 (8.2–19.9)	0.7 (0.0–2.2)
positive ^1^	≥ 17	309 (8%) (-)	53.4 (47.8–59.0)	13.3 (9.5–17.1)	33.3 (28.1–38.6)	6.1 (3.5–8.8)

1 shown for reference, not used in the simulation. More detailed results are reported in reference [7]Abbreviations: FIT, fecal immunochemical test; Hb, hemoglobin.

### Modelling outcomes

**Fig. 1:**
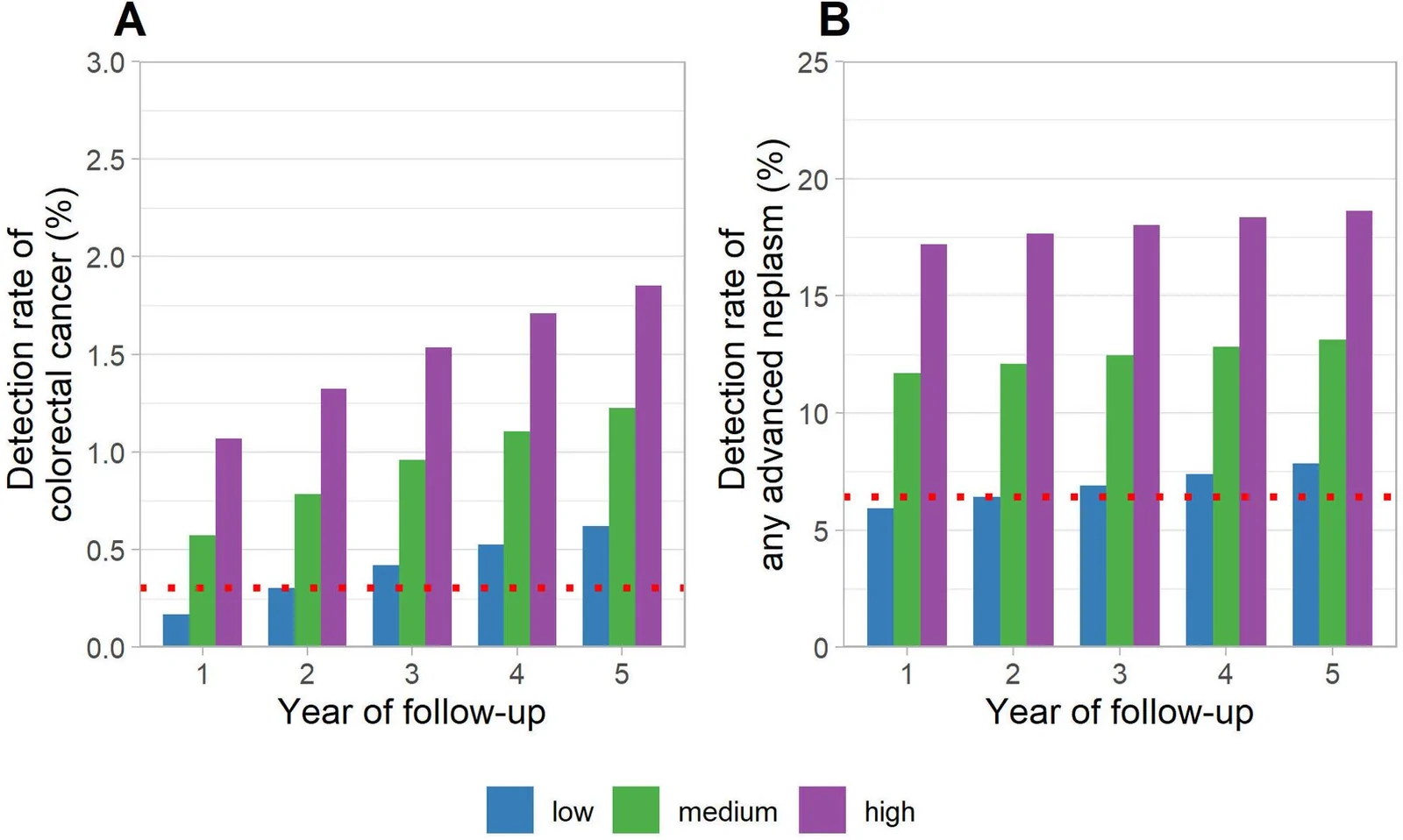
Expected detection rates of (**A**) colorectal cancer and (**B**) any advanced neoplasm at 1-5 years of follow-up by FIT-negative subgroup. FIT negative subgroups (FOB Gold, Sentinel Diagnostics): low <8; medium 8-<10; high 10-<17 µg Hb/g stool. Dotted line (benchmark): detected cancers (panel A) and any advanced neoplasms (panel B) in low-negative subgroup 2 years after initial screening (a commonly recommended screening interval). Any advanced neoplasm: cancers and advanced adenomas; FIT, fecal immunochemical test

In the simulated cohorts, detection rates of cancers and any advanced neoplasms over time differed markedly by FIT-negative category (Fig. 1**)**. Cancer detection rates were 0.17%, 0.30%, 0.42%, 0.53%, and 0.62%, for year 1–5 after negative index screening, respectively, in the low-negative subgroup. This compared to detection rates of 0.57%, 0.78%, 0.96%, 1.10% and 1.22% for medium-negative and 1.07%, 1.33%, 1.53%, 1.71%, and 1.85% for high-negative subgroups, for year 1–5, respectively. Put another way, detection rates in the low negative subgroup were between 49 and 70% lower than those in medium-negative patients, and between 67 and 84% lower than those in high-negative patients. The pattern was similar for any advanced neoplasms.

With respect to the time points corresponding to currently recommended screening intervals, i.e., year 1 and 2 of follow-up, detection rates among medium- and high-negative subgroups exceeded those among low-negative patients by far. Cancer detection rates for medium- and high-negative subgroups at year 1 were 1.9- to 3.5-fold higher than those for low-negatives at year 2 (dotted red line in Fig. 1).

**Fig. 2:**
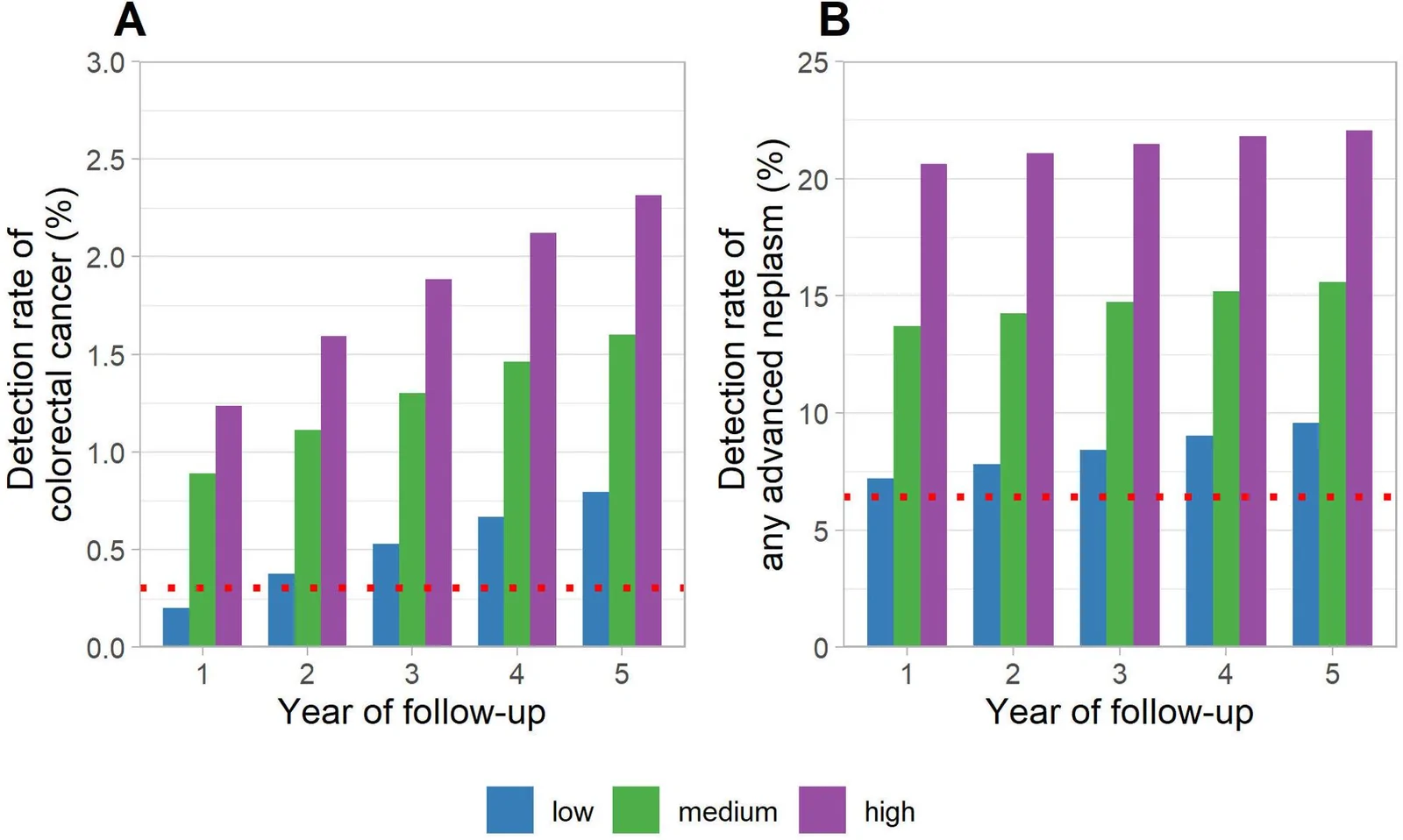
Expected detection rates of (**A**) colorectal cancer and (**B**) any advanced neoplasm 1-5 years of follow-up by FIT-negative subgroup, men. FIT negative subgroups (FOB Gold, Sentinel Diagnostics): low <8; medium 8-<10; high 10-<17 µg Hb/g stool. Dotted line (benchmark): detected cancers (panel A) and any advanced neoplasms (panel B) in low-negative subgroup 2 years after initial screening (a commonly recommended screening interval). Any advanced neoplasm: cancers and advanced adenomas; FIT, fecal immunochemical test

**Fig. 3:**
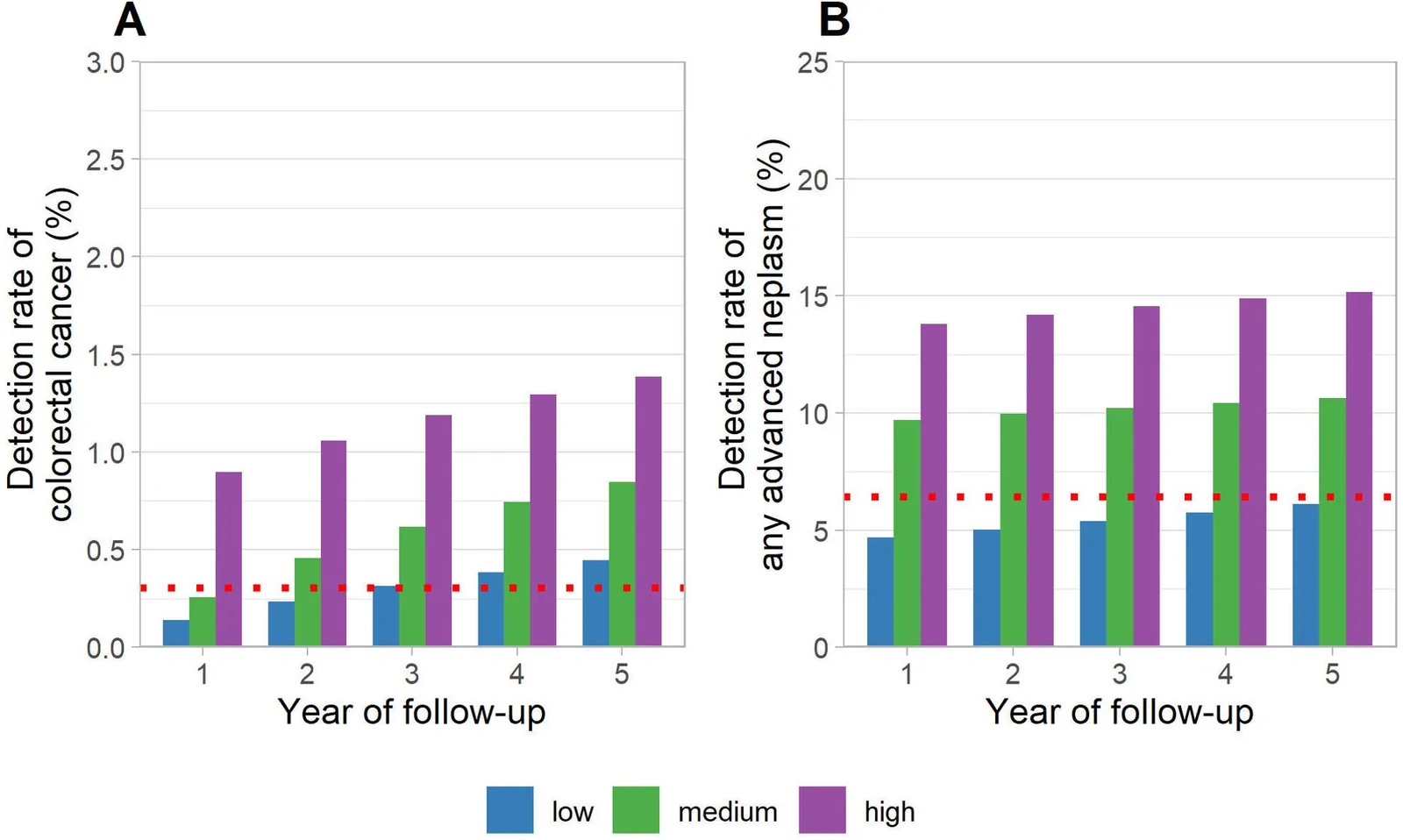
Expected detection rates of (**A**) colorectal cancer and (**B**) any advanced neoplasm at 1-5 years of follow-up for all patients with negative FIT and by FIT-negative subgroup, women. FIT negative subgroups (FOB Gold, Sentinel Diagnostics): low <8; medium 8-<10; high 10-<17 µg Hb/g stool. Dotted line (benchmark): detected cancers (panel A) and any advanced neoplasms (panel B) in low-negative subgroup 2 years after initial screening (a commonly recommended screening interval). Any advanced neoplasm: cancers and advanced adenomas; FIT, fecal immunochemical test

Sex-specific analyses revealed substantially higher detection rates among men as compared to women across all FIT-negative subgroups (Fig. 2 and Fig. 3). However, the patterns seen in the overall population were consistent in sex-specific strata, with cancer detection rates among medium- and high-negative patients considerably higher as compared to those in the low-negative subgroup. Cancer detection rates in women in the medium-negative subgroup at year 1 were slightly lower than the 2-year prevalence of all low-negative patients but exceeded that level at year 2 of follow-up.

**Fig. 4:**
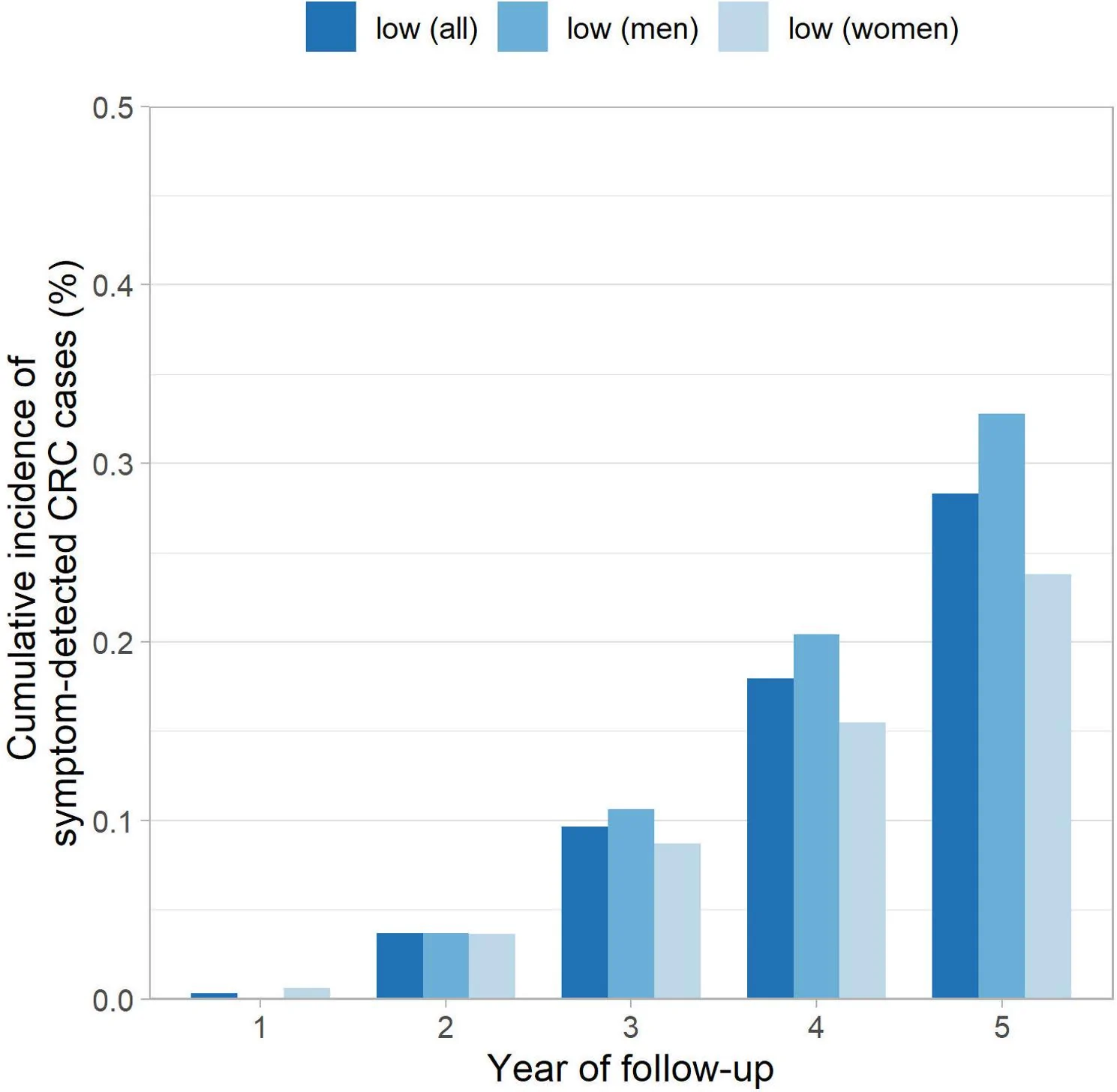
Cumulative interval cancer incidence in low-negative FIT participants. CRC, colorectal cancer; FIT, fecal immunochemical test

Finally, the expected cumulative interval cancer incidence of among participants with low-negative FIT values remained very low (below 0.04%) within two years of follow-up, but would increase to close to 0.3% if the screening interval extended up to 5 years (Fig. 4).

## Discussion

This modelling study explored the potential design of risk-adapted annual and biennial FIT screening intervals based on risk stratification among individuals with a negative FIT. Individuals were grouped into low, medium, and high negative subgroups according to their hemoglobin levels at FIT screening and then followed up over time. After 1–5 years of follow-up, expected cancer detection rates among the small groups of medium- and high-negative patients were up to six times higher than in the large group of low-negative patients, and expected detection rates of cancer or any advanced neoplasia for medium- and high-negative patients exceeded the expected 2-year detection rates of the low-negative group already after 1 year of follow-up. Sex-specific analyses showed consistent patterns but revealed overall lower prevalences in women versus men. The expected incidence of symptomatic CRC would be very low within two years after a low-negative FIT, but would increase quite substantially with longer follow-up intervals. Taken together, these results suggest strong potential of quantitative FIT values for risk stratification and defining risk-adapted screening intervals, such as one year for the small group of participants with medium- and high-negative fecal hemoglobin concentrations, and two years for the vast majority of participants with low-negative fecal hemoglobin concentrations.

### Findings in context

This study contributes twofold to previous literature. First, as previously pointed out [7], our analyses of BLITZ add to the body of evidence that those in the upper range of FIT negative hemoglobin values are at higher risk of developing CRC as compared to those in the lower range [31–33]. In contrast to previous studies, all patients in BLITZ underwent colonoscopy, implying that most advanced neoplasms will have been detected, including those in individuals with a negative FIT.

Second, our study substantiates previous calls for risk-adapted FIT screening intervals by deriving expected risk-group specific neoplasia detection rates over time. To our knowledge, no longitudinal evidence on expected outcomes by risk-groups based on stool hemoglobin levels has been published. Previous proposals on risk-adapted FIT intervals considering the hemoglobin level were based on the prevalences of neoplasm findings after first or repeated FIT screening but lack follow-up data [7, 31–33]. The combination of baseline neoplasm prevalences by risk group along with projected cancer and advanced neoplasm detection rates over time enhances the evidence base to inform on risk-adapted FIT screening intervals. Of note, the currently ongoing Dutch PERFECT‑FIT trial will be the first longitudinal study to provide evidence on the effectiveness of personalized CRC screening through tailored invitation intervals of 1–3 years based on prior hemoglobin concentrations [34].

Our findings illustrate that current FIT screening approaches in most major healthcare systems, regardless of applied cut-off, could be enhanced by allocating individuals according to their hemoglobin level at previous screening to annual or biennial screening. Biennial intervals, as currently uniformly recommended in several advanced healthcare systems, such as Germany and the Netherlands, will mostly be appropriate for the large group of patients with ‘low-negative’ hemoglobin concentrations. In our study, individuals in this group had considerably lower CRC risks even after 2 years of follow-up as compared to individuals in the medium- or high-negative group. This is reassuring, given this subset represents 90% of all FIT negative patients at first screening.

Annual intervals, in contrast, may mostly be appropriate for the few individuals with elevated FIT hemoglobin concentrations, even within the negative range. In our study, medium- or high-negative individuals were at up to 6-fold increased CRC risks compared to individuals with lower FIT values. While these groups represent only ~ 10% of all FIT-negative patients at previous screening, systematically referring medium- or high-negative individuals to biennial instead of annual intervals implies strong potential for harm, as earlier screening might have spared patients a cancer diagnosis. For healthcare systems uniformly applying biennial screening, this may be viewed as a false-negative recommendation (i.e., recommending a longer when in fact a shorter interval is appropriate) in 1 out of 10 individuals, which is of major concern given the population-based nature of CRC screening.

Our findings also imply that countries with uniform recommendations for annual screening, such as the US, might be prone to significant over-screening, as large shares of individuals will be recommended annual screening while biennial screening would be more appropriate. This may be viewed as a false-positive recommendation in 9 out of 10 individuals (i.e. recommending a shorter when in fact a longer interval is appropriate). False-negative recommendations constitute the more substantial error, and avoiding them should be given higher priority in designing offers. However, false-positive recommendations on the scale of a population-wide screening represent a likewise worrisome finding, as they subject individuals to a burdensome intervention and add to cost and capacity constraints in the health care system. In healthcare systems with uniform recommendations for annual screening, screening intervals could therefore possibly be extended to two years for most individuals with negative FIT at previous screening.

Using risk-adapted FIT screening intervals, it may be expected that among those allocated to shorter intervals due to higher hemoglobin concentrations, the share of false-positive tests will increase, implying partially unnecessary colonoscopies [34]. However, given that avoiding false negatives should be given higher priority, as discussed above, we expect the balance between potential benefits and harms of risk-adapted screening to be strongly positive.

Finally, as our supplementary analyses show, low detection cancer rates in low-negative patients at 1- or 2-year follow-up are likely not attributable to interval cancers. However, the cumulative incidence of interval cancers steadily increases over time, which challenges previous considerations to prolong FIT screening intervals beyond 2 years [7, 31–33]. Yet, based on consistent findings in many previous CRC screening studies, it is generally accepted that most individuals will be inherently at low CRC risk. The large majority of screening participants will be without any polyp finding, even at repeat screening [35–37]. Further study on potential risk stratification allowing for intervals > 2 years is therefore needed.

### Public health considerations

Risk stratification for CRC screening is an area of major interest as so far very few factors have been identified with strong discriminatory power (e.g., family history). Apart from hemoglobin level-based stratification, approaches involve algorithms based on genetic or environmental risk scores [38]. However, efficacy in ideal setting, including modelling studies, might vary considerably from real-life effectiveness. Specifically, highly efficacious strategies based on sophisticated approaches might be impractical to implement and could even harm screening participation, resulting in lower overall impact [4]. Therefore, caution must be exercised when considering changes to population-wide screening recommendations.

Yet, our results suggest that risk stratification by negative FIT values may be a suitable candidate to improve screening outcomes. As quantitative hemoglobin concentrations are routinely available in FIT-based screening programs, such risk stratification would be possible without any extra efforts of collecting biological samples or data collection and processing. In screening practice, patients would still undergo the same procedures as with the currently implemented uniform approach. For those with negative FIT, the major difference would lie in the recommended interval for re-screening and associated patient communication, an increased level of complexity in program management likely manageable in major health care systems, specifically as the decision problem is only binary (i.e., annual or biennial interval). Finally, as only approximately 10% of patients would be directed to annual FIT re-screening, the impact on colonoscopy capacities will likely be limited in systems currently using uniform biennial intervals, and even capacity saving in systems currently using annual intervals.

We deliberately chose a non-sex specific approach as main analysis as current recommendations are also insensitive to sex, and further stratification factors would add to program complexity. However, sex-stratified analyses revealed deviation both in terms of neoplasm prevalences after first negative FIT, as well as in terms of expected cancer and advanced neoplasm detection rates over time. Further studies are needed to explore potential for further risk stratification with easily obtainable factors such as sex and age.

### Limitations

Specific limitations of COSIMO have been described previously [8]. Briefly, major limitations concern simplifying model assumptions and uncertainties related to input parameters. For example, the German national screening colonoscopy registry, the key data source to derive transition rates between stages, did not include sufficiently detailed data to calculate specific transition rates for proximal and distal neoplasms. Therefore, no subsite-specific analysis can be conducted using the model.

Our analyses are limited by the partly small sample size of FIT negative subgroups in the BLITZ study, which translates to higher uncertainty of the input parameters to the simulation model. A further limitation is the lack of further factors suitable for risk stratification, such as age or environmental and genetic information. Regarding age, even the very large BLITZ cohort was too small to allow for appropriate age-specific analyses within FIT-negative subgroups. Thus, the model starting age was set to 60 years, in line with median age of the included BLITZ cohort. However, as adenoma prevalences increase with age and screening recommendations typically start age 45 or 50, the potential for risk-adapted screening intervals may vary by age, which could not be assessed in this study.

As there is limited evidence on repeat screening outcomes, in this study, risk-stratification was considered only for one screening round whereas FIT needs to be performed repeatedly to be effective. Patterns of hemoglobin concentrations and prevalences may change over several rounds of screening. However, hemoglobin concentration is a good predictor of advanced neoplasia [39], an association unlikely to be affected by repeated screening. As risk increases by age, in a repeat screening setting of a given birth cohort, the share of patients allocated to shorter screening intervals may gradually increase over time. Further evidence from real-life studies will be needed to assess implications of repeated screening.

As well, the risk-group specific cancer and adenoma baseline prevalences used in our model were informed by the BLITZ study based on FIT data collected via the Sentinel FOB Gold which may challenge generalizability. However, the diagnostic performance of the Sentinel FOB Gold was previously shown to be comparable to those of other commonly used FITs, such as the OC Sensor [30], and patterns of risk-group-specific prevalences seen in BLITZ correspond to those in other study collectives [31–33]. Additional evidence should be collected for varying populations and FIT testing kit to further substantiate our findings. Finally, cost-effectiveness considerations, including metrics such as costs per quality-adjusted life years were beyond the scope of this study and should be addressed in future research.

### Conclusions

Fecal hemoglobin concentrations among FIT negative participants may be a readily available, highly informative tool for defining risk-adapted annual or biennial FIT screening intervals.

## Supplementary Information

Below is the link to the electronic supplementary material.

Supplementary Material 1: Additional File 1: Supplementary Methods; Tables S1-S4; Figure S1-S2

## Data Availability

All analyses relevant to the study are included in the article or uploaded as supplementary information. The model source code is available from https://www.dkfz.de/en/klinepi/download/index.html. Further information is available from the corresponding author upon request.

## Abbreviations

CRC: Colorectal cancer
DKFZ: German Cancer Research Center (Deutsches Krebsforschungszentrum)
FIT: Fecal Immunochemical Test
Hb: Hemoglobin
US: United States
BLITZ: Begleitende Evaluierung innovativer Testverfahren zur Darmkrebs-Früherkennung (engl.: Accompanying evaluation of innovative test procedures for the early detection of colorectal cancer)
COSIMO: Colorectal Cancer Multistate Simulation Model
PERFECT-FIT: Personalized colorectal cancer screening based on prior fecal hemoglobin concentration in a FIT-based screening program (ongoing Dutch trial)
